# Research advances in evaluation methods for neoadjuvant therapy of tumors

**DOI:** 10.3389/fonc.2025.1580360

**Published:** 2025-05-22

**Authors:** Zien Yuan, Ting Chen, He Zhang, Jiatong Li, Juntan Li, Guanning Shang

**Affiliations:** Department of Orthopedics, Shengjing Hospital of China Medical University, Shenyang, China

**Keywords:** tumors, neoadjuvant therapy, evaluation method, precision medicine, review

## Abstract

Preoperative neoadjuvant therapy is crucial for large malignant tumors or tumors that are challenging to resect. Consequently, an objective assessment of its therapeutic efficacy is important. Currently, the conventional evaluation methods for neoadjuvant therapy of tumors are mainly divided into two categories: imaging-based and pathological evaluations. In imaging-based evaluation, the World Health Organization criteria are straightforward; however, they exhibit some issues such as unclear criteria for minimum lesions and measurement errors. Moreover, although the Response Evaluation Criteria In Solid Tumors criteria have been improved, they remain insensitive to internal tumor changes and are prone to measurement errors. The Modified Response Evaluation Criteria In Solid Tumors criteria are specifically designed for hepatocellular carcinoma, yet they have limitations, such as difficulty defining complex tumor boundaries. The Positron Emission Tomography Response Criteria in Solid Tumors criteria, which integrate positron emission tomography/computed tomography, offer high accuracy but are influenced by factors related to the patient’s body condition and equipment. The Choi criteria, which comprehensively consider tumor size and density, can be used to evaluate the efficacy of targeted therapy; however, they are characterized by cumbersome measurement procedures and strong subjectivity. In terms of pathological evaluation, the Huvos score determines the therapeutic effect based on the degree of tumor necrosis, which can guide subsequent treatment and prognosis. However, the evaluation time is fixed and subject to interference from pathological procedures. The Miller–Payne criteria focus on changes in the number and density of tumor cells and provide a reference for surgical decision-making. Nevertheless, it does not consider lymph node metastasis. The Residual Cancer Burden assessment criteria comprehensively quantify residual tumors by integrating multiple factors. Moreover, these offer a precise assessment of breast cancer and have a high value in predicting prognosis. However, their parameter calculation is complex and highly subjective. In summary, each method has its own advantages and disadvantages. With the advancement of scientific research, evaluation methods for neoadjuvant therapy are constantly evolving. In-depth research into these methods can help identify more accurate and effective evaluation strategies, providing a more scientific basis for tumor treatment and propelling the field of tumor therapy toward greater precision.

Neoadjuvant therapy for tumors, which is a crucial component of comprehensive tumor treatment, has been increasingly applied in clinical practice in recent years (1–3). Neoadjuvant therapy aims to administer systemic or local treatments before surgery or other local interventions. Its goals are to reduce tumor volume, downstage the tumor, increase the surgical resection rate, and improve the prognosis of patients (4). With continuous advancements in medical technology, the methods of neoadjuvant therapy for tumors have become increasingly diverse, encompassing chemotherapy, radiotherapy, targeted therapy, immunotherapy, and others. However, the accurate evaluation of the effectiveness of neoadjuvant therapy has emerged as a significant issue for clinicians and researchers. An accurate evaluation method can not only assist doctors in promptly adjusting treatment regimens to enhance treatment efficacy but also provide patients with more personalized treatment recommendations, thereby improving patients’ quality of life and survival prognosis (5). Currently, the commonly used evaluation methods for neoadjuvant therapy in clinical practice mainly include imaging examinations and pathological evaluations. Each of these methods has its own advantages and disadvantages, and their application effects vary among different types of tumors and treatment regimens. Therefore, an in-depth study of the research progress in evaluation methods for neoadjuvant therapy holds great clinical significance for optimizing tumor treatment strategies and improving the treatment outcomes for patients. This article aims to review the research progress in evaluation methods for neoadjuvant therapy to provide references for clinical practice and scientific research.

## 1 Imaging-based tumor evaluation methods

### 1.1 World health organization criteria

**Figure 1:**
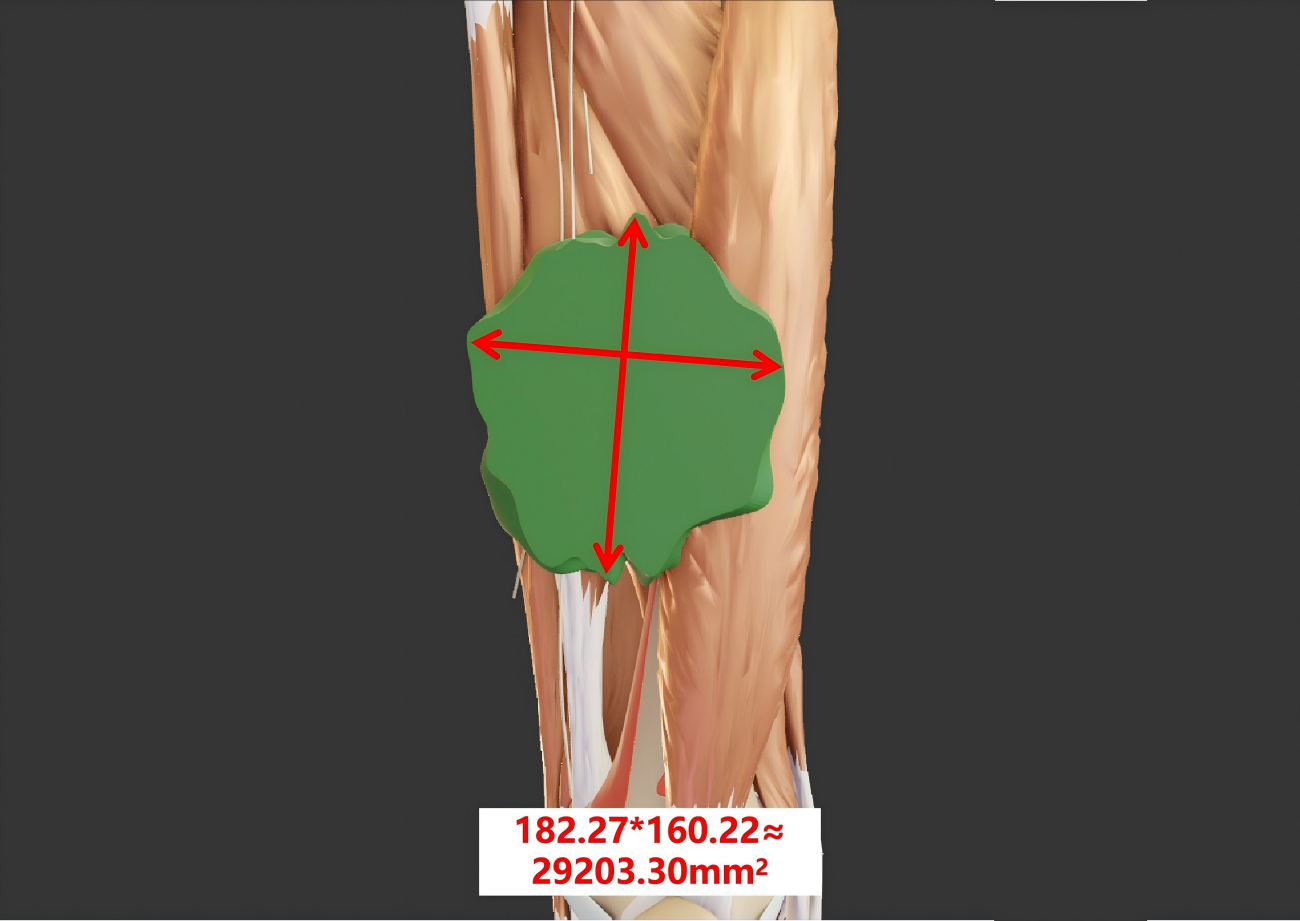
The tumor size is evaluated according to the WHO criteria. The longest diameter of the tumor measures 182.27 mm, while the longest perpendicular diameter measures 160.22 mm. The product of these two diameters is 29,203.30 mm².

In 1981, the WHO established the first evaluation criteria for tumor efficacy, namely, the WHO criteria (6). These criteria use the change in the product-sum of the longest diameter of the tumor lesion and its longest perpendicular diameter to assess the therapeutic effect (7) (**Figure 1**). Based on the degree of tumor change, tumors are classified into four grades: a. complete response (CR): the tumor-free state persists for at least 4 weeks after treatment; b. partial response (PR): the tumor volume is reduced by ≥50% compared with that before treatment, and this reduction persists for at least 4 weeks; c. stable disease (SD): the tumor volume reduction does not meet the criteria for PR, and the increase does not meet the criteria for progressive disease (PD); d. PD: in one or more lesions, the tumor volume increases by ≥25% compared with that before treatment.

The calculation method for tumor treatment evaluation according to the WHO criteria is relatively simple. It requires measuring the two largest perpendicular diameters of the tumor and calculating their product. This method is easy to master and apply without the need for complex techniques or equipment support. Moreover, the WHO criteria have been applied in the field of tumor treatment evaluation for a long time, accumulating a vast amount of clinical data and experience. Many clinical studies and practices have adopted this criteria, providing a degree of comparability among different studies.

However, the WHO criteria have some drawbacks. They do not clearly define the minimum size and quantity of lesions to be measured. Regarding specific measurements and calculations, because the WHO criteria use the product of the two largest perpendicular diameters of the tumor as the evaluation standard, slight changes in tumor size or measurement errors can significantly affect the final assessment results. Tumors often have irregular shapes, and the WHO criteria cannot comprehensively reflect three-dimensional morphological changes. When necrosis occurs within a tumor, the tumor volume may not change significantly, and the WHO criteria may overlook this point, leading to errors in the evaluation of therapeutic efficacy.

### 1.2 Response evaluation criteria in solid tumors criteria

#### 1.2.1 RECIST version 1.0

RECIST 1.0 was proposed by the National Cancer Institute of the United States in 2000. These criteria have evolved from the WHO criteria. RECIST 1.0 assesses the size of tumors by the sum of the longest diameters of tumors and retains the tumor change grading of the WHO criteria (8), which is as follows: a. CR: the tumor-free state persists for at least 4 weeks after treatment; b. PR: the sum of the longest tumor diameters is reduced by ≥30% compared with that before treatment, and this reduction persists for at least 4 weeks; c. SD: after treatment, the sum of the longest tumor diameters does not meet the criteria for PR, and the increase does not meet the criteria for PD; d. PD: the sum of the longest tumor diameters increases by ≥20% compared with that before treatment.

RECIST 1.0 introduced new imaging techniques, such as computed tomography (CT) and magnetic resonance imaging (MRI), and clearly defined the measurement methods and standards for tumor lesions shown by these imaging techniques. It specifies the minimum size of the lesions to be measured: the maximum diameter of a tumor lesion should be ≥20 mm on X-ray films and ≥10 mm on CT examinations. RECIST 1.0 also specifies the number of target lesions to be measured, with a maximum of 10 throughout the body and up to 5 in the same organ. By measuring the sum of the longest diameters of tumors, RECIST 1.0 simplified the measurement process compared with the WHO criteria, reducing measurement errors and subjectivity.

#### 1.2.2 RECIST version 1.1

RECIST 1.1 was proposed in 2009. Compared with version 1.0, it improved the efficacy evaluation criteria. For CR, in addition to the disappearance of all lesions, it added that the short-axis value of any pathological lymph node (regardless of whether it is a target lesion) must be <10 mm. Regarding PD, based on the sum of the longest diameters of the minimum target lesions recorded after the start of treatment, the sum of the longest diameters of the target lesions should increase by at least 20%, and the increase in the sum of the longest diameters should be at least 5 mm or new lesions should appear. In addition, the criteria for measuring target lesions specified in RECIST 1.1 have been refined: a maximum of five target lesions can be measured across the body, with no more than two in the same organ.

RECIST 1.1 clearly defined the measurement criteria for pathological lymph nodes, using the short-axis value as the standard. Lymph nodes with a short-axis value of <10 mm are considered normal lymph nodes, and those with a short-axis value of 15 mm or more are defined as pathological lymph nodes to be measured. For lymph nodes with a short-axis value between 10 and 15 mm, clinical judgment is required to determine whether they should be measured (9). Unlike the old version, in which the appearance of new lesions was immediately judged as disease progression, the new version is more cautious in assessing newly developed lesions. A comprehensive assessment in combination with clinical conditions is required to determine whether the newly developed lesions represent tumor progression. Having been improved from RECIST 1.0, it has higher requirements for measurement accuracy and consistency in practical operations, and it is now the most widely used evaluation method for solid tumors (9).

**Figure 2:**
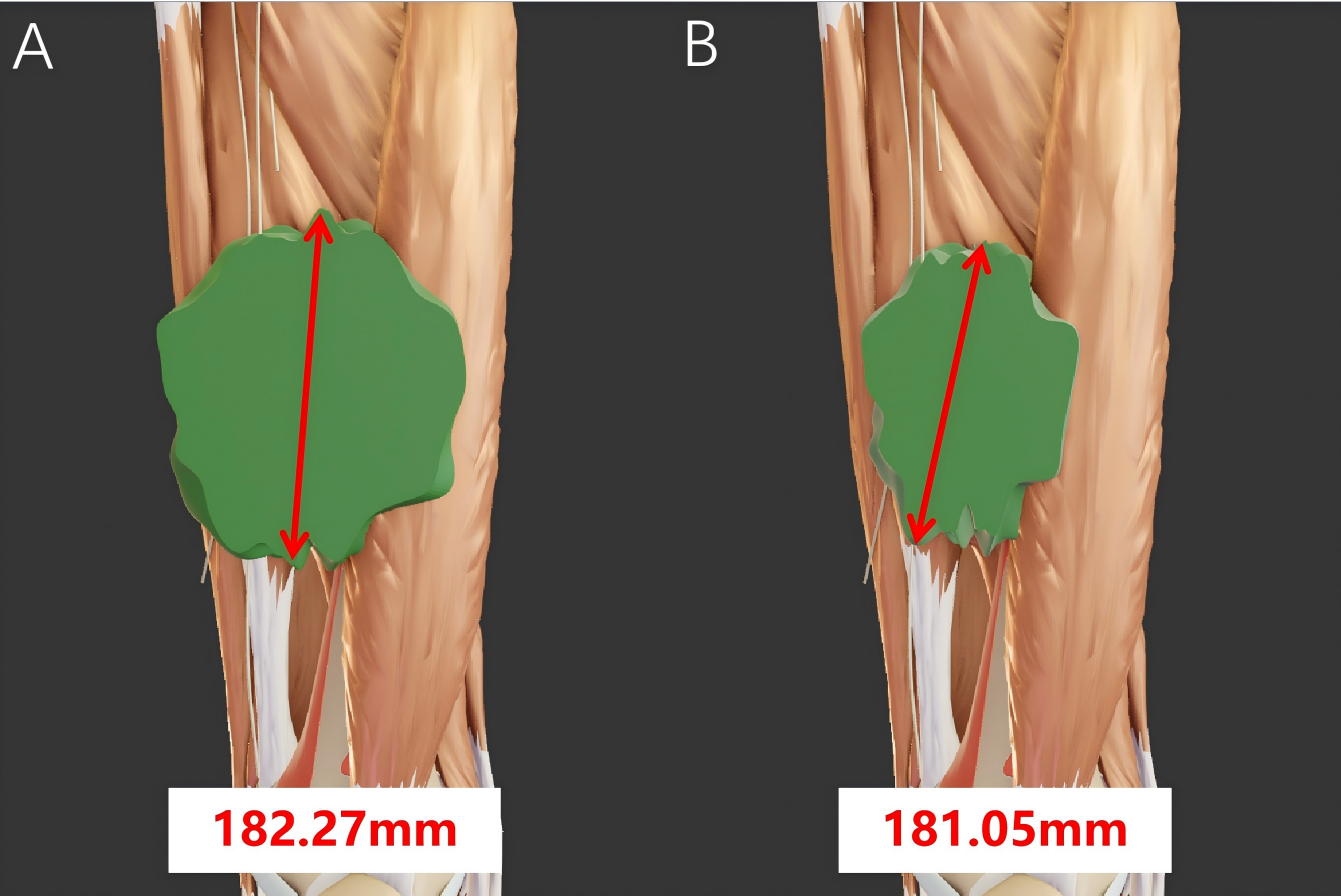
The tumor size before and after neoadjuvant therapy was calculated according to the RECIST criteria. **(A)** The longest diameter of the tumor measures 182.27 mm before neoadjuvant therapy and **(B)** 181.05 mm after neoadjuvant therapy. The tumor mainly shrinks along the short diameter, resulting in a significant reduction in volume. However, there is no notable change in its longest diameter. In this case, misjudgment may occur when using the RECIST criteria.

The limitations of RECIST 1.1 mainly lie in its continued reliance on the evaluation of therapeutic efficacy based on two-dimensional size changes of the entire tumor. Cells in different regions within the tumor may respond differently to treatment, with varying regression rates, leading to nonconcentric regression and formation of irregularly shaped tumors (10). As a result, RECIST 1.1 may misjudge the treatment effect (**Figure 2**). Furthermore, in some cases, although the morphological size of a tumor may not change significantly, significant alterations may have occurred in the biological characteristics of the tumor cells, such as metabolism and invasiveness. However, RECIST 1.1 is unable to capture these changes in a timely manner. For instance, in targeted therapies, where the primary therapeutic effect is to stabilize tumor cells, the regression of tumor volume does not directly reflect the treatment effect (11). If internal tumor bleeding occurs, the tumor volume may even show an increasing trend (12). In such cases, RECIST 1.1 may not fully reflect the unique efficacy characteristics of targeted therapies, necessitating a comprehensive assessment in combination with other indicators. In addition, RECIST 1.1 mainly measures tumor size through CT. However, there is a relatively large error in tumor size measurements by different individuals, and even measurements of the same tumor by the same person on two occasions can vary significantly (13).

### 1.3 Modified response evaluation criteria in solid tumors criteria

Given the inaccuracy of assessing tumors treated with targeted therapy solely based on size (14, 15), the European Association for the Study of the Liver proposed the modified RECIST criteria, namely, the mRECIST criteria, for patients with hepatocellular carcinoma in 2010 (16). The mRECIST criteria classify tumors into four grades: a. CR: all lesions enhanced in the arterial phase disappear after treatment; b. PR: the sum of the diameters of lesions enhanced in the arterial phase is reduced by ≥30% compared with that before treatment; c. SD: the sum of the diameters of lesions enhanced in the arterial phase does not decrease enough to meet the PR criteria or increase enough to reach the PD criteria after treatment; d. PD: the sum of the diameters of lesions enhanced in the arterial phase increases by ≥20% compared with that before treatment or new lesions larger than 1 cm appear. These criteria emphasize that parts of the lesions that enhance in the arterial phase of contrast-enhanced CT or contrast-enhanced MRI should be regarded as viable tumors, and the volume of viable tumors should be calculated (17). Compared with RECIST 1.1, the mRECIST criteria exhibit no differences in the minimum size and quantity of the lesions to be measured. There is only a slight change in the measurement method. RECIST 1.1 measures the maximum diameter of the lesions, whereas the mRECIST criteria measure the maximum diameter of the enhanced part within the lesions.

The mRECIST criteria, which only consider the size changes of viable tumors, have improved accuracy compared with the traditional RECIST criteria. The mRECIST criteria are primarily used for efficacy evaluation in patients with hepatocellular carcinoma (18, 19). However, recent studies have shown that the mRECIST criteria are effective in assessing the treatment outcomes of different types of cancers, including malignant pleural mesothelioma and non-small cell lung cancer (20, 21).

For some tumor cells that become complex after treatment, inflammatory reactions, fibrotic tissue hyperplasia, and residual viable tumor cells may be intertwined. In this case, the mRECIST criteria do not define an objective standard to demarcate the boundaries of viable tumors. For tumors that increase in volume at the early stage of treatment due to internal inflammatory reactions or immune cell infiltration, the mRECIST criteria may still misjudge this as disease progression.

### 1.4 Positron emission tomography response criteria in solid tumors criteria

In 2009, in response to the unique characteristics of certain neoadjuvant therapies that inhibit tumor cell biological activity rather than directly killing them, Wahl et al. proposed a new standard for evaluating tumor treatment efficacy in combination with PET-CT based on the RECIST evaluation criteria, namely, the PERCIST criteria (22). During the evaluation process, the PERCIST criteria incorporate the fluorodeoxyglucose (FDG) metabolic imaging information from PET-CT. These criteria consider factors such as the standardized uptake value corrected for lean body mass (SUL), peak standardized uptake value corrected for lean body mass (SULpeak), and total lesion glycolysis. According to these criteria, tumors are classified into four grades: a. CR: after treatment, the FDG metabolism of all lesions is lower than that of the arterial reference area, with no macroscopically visible lesions showing increased metabolism, and no new lesions appear; b. PR: after treatment, the SULpeak of the lesions decreases by at least 30%, and the absolute value of SUL decreases by at least 0.8; c. SD: conditions do not meet the criteria for CR, PR, and PD; d. PD: after treatment, the SULpeak of the lesions increases by at least 30% and the absolute value of SUL increases by at least 0.8. In addition, the total lesion glycolysis of the lesions increases by 75% or new lesions appear. The PERCIST criteria do not explicitly define the minimum lesion size for measurement. However, to ensure a reliable standardized uptake value (expressed as SUL), the lesion should have a diameter of ≥1 cm and demonstrate FDG uptake across at least three consecutive slices. The PERCIST criteria do not strictly limit the number of lesions that can be measured. However, the preferred approach is to use the single most metabolically active lesion, defined by the highest peak SUL value, as the primary focus of evaluation.

The PERCIST criteria focus on changes in tumor metabolic activity. Even when there are no obvious changes in tumor morphology, the efficacy of tumor treatment can be evaluated based on metabolic changes. Clinical studies have shown that the PERCIST criteria are more accurate in assessing tumor efficacy than other criteria, such as the WHO and RECIST criteria (23, 24). The PERCIST criteria also provide clear guidelines regarding the acquisition, processing, and analysis of PET images, including the imaging time after injecting the imaging agent, method of delineating the region of interest, and setting of the SUL threshold, thereby improving the consistency and reproducibility of the evaluation results (25). The PERCIST criteria allow for simultaneous evaluation of metastatic lesions, providing a more comprehensive understanding of changes in tumor burden. As long as tumors with metabolic activity can be observed through PET imaging, efficacy can be evaluated according to the PERCIST criteria. These criteria are applicable to various cancers, such as lung, breast, colorectal, and pancreatic cancers, demonstrating good versatility and adaptability (26–29).

However, during tumor treatment, the body may experience an inflammatory reaction, which can increase tissue metabolic activity, potentially mimicking the manifestations of tumor recurrence or progression. This can complicate the accurate assessment of disease status using the PERCIST criteria. Infected lesions may also take up the PET tracer, especially in patients with immunodeficiency, in whom the incidence of infection is relatively high, further complicating the evaluation using the PERCIST criteria. Although 18F-FDG has a certain affinity for tumor cells, some normal tissues with high metabolic activity, such as the brain and myocardium, can also take up the tracer, which may lead to confusion with tumors. Finally, PET-CT devices produced by different manufacturers or models may have variations in spatial resolution and sensitivity, which could interfere with the final prediction results.

### 1.5 Choi criteria

In 2007, Choi et al. from the MD Anderson Cancer Center first proposed new evaluation criteria for neoadjuvant therapy of tumors, namely, the Choi criteria (30). The research team found that when imatinib was used to treat gastrointestinal stromal tumors, owing to the special treatment features of targeted drugs that mainly stabilize tumor cells, the tumor volume often showed no significant change in the early treatment stage. At this time, the RECIST criteria often underestimated the efficacy of targeted drugs. Therefore, Choi et al. proposed the Choi criteria, which divides efficacy into four criteria: a. CR: all lesions disappear and no new lesions appear; b. PR: the longest diameter of the tumor measured by CT shrinks by >10% or the tumor density decreases by >15%, and there are no new lesions or obvious progression of unmeasurable lesions; c. SD: the tumor does not meet the criteria of CR, PR, or PD, and there is no symptom deterioration caused by tumor progression; d. PD: the longest diameter of the tumor increases by ≥10% and the density change does not meet the PR criteria. New lesions appear, there is an increase in the size or volume of the original intratumoral nodules. The Choi criteria do not explicitly define the minimum lesion size for measurement; however, the lesions must meet the CT value measurability requirement. The lesions should include a sufficient number of slices to calculate density changes, typically aligning with the RECIST criteria, which require the longest diameter of ≥10 mm. The Choi criteria do not specify the number of lesions to be measured. Multiple lesions may be assessed, but changes in CT values must be evaluated individually.

Compared with the RECIST criteria, the Choi criteria consider both the size and density of the tumors. In particular, when targeted drugs are used, the Choi criteria can more objectively reflect the treatment effects. For tumors whose volume remains unchanged during treatment but whose internal density decreases, the Choi criteria can provide more timely evaluation compared with the RECIST criteria, which may lag in such cases. When the Choi criteria were first proposed, they were mainly applied for the efficacy evaluation of gastric stromal tumors. However, similar changes may also occur during the treatment of other tumors. Currently, the Choi criteria are applied for the efficacy evaluation of tumors such as colorectal cancer and soft-tissue sarcomas (31, 32), and their scope of use is expected to become even broader in the future. The Choi criteria can also enhance the ability to predict patient prognosis. Patients who are judged to have a favorable treatment response according to the Choi criteria tend to have longer progression-free survival and overall survival, providing important reference value for the prognosis of patients.

Although the Choi criteria demonstrate significant advantages over the RECIST criteria, they have certain limitations. The Choi criteria require measuring both the size and density changes of tumors, which is a relatively cumbersome process. The CT values, which serve as the primary basis for evaluation, cannot be directly obtained by doctors through simple film reading, thereby increasing the complexity of the evaluation. Moreover, when measuring tumor density, different doctors may judge the degree of decrease based on their own experience, thereby increasing the subjectivity of the evaluation. Finally, the Choi criteria were initially proposed to evaluate the efficacy of neoadjuvant treatment for gastrointestinal stromal tumors, and the value of using them to evaluate the efficacy of treating other tumors remains to be explored. Above mentioned imaging-based tumor evaluation methods could been seen in **Table 1**.

**Table 1:** Basic information, advantages, and disadvantages of imaging-based evaluation methods.

Evaluation criteria	Proposed time	Measurement method	Grading criteria	Advantages	Disadvantages
WHO Criteria	1981	Product of the maximum longitudinal diameter and the maximum perpendicular diameter of the tumor	CR, PR, SD, PD	Evaluation method is simple	For tiny tumors, it is difficult to accurately measure their longest diameter and the longest perpendicular diameter, which in turn affects the accuracy of the evaluation; it cannot reflect the three-dimensional changes of tumors
RECIST Criteria	2000	Sum of the maximum longitudinal diameters of the tumor	CR, PR, SD, PD	Define the measurement criteria for lymph nodes; simplify the measurement process	It cannot reflect the three-dimensional morphological changes of the tumor as well as the inactivated and necrotic tissues inside the tumor
mRECIST Criteria	2010	Maximum diameter of the enhanced part within the tumor	CR, PR, SD, PD	Only consider the size changes of viable tumors and can provide more accurate evaluation	It is unable to evaluate the interwoven situation between viable tumors and surrounding tissues
PERCIST Criteria	2009	Uptake of 18F-FDG in the tumor	CR, PR, SD, PD	Evaluating the curative effect through tumor metabolic changes is more accurate	Inflammation, infection, or tissues with inherently high metabolic activity can cause interference
Choi Criteria	2007	Percentage change in the longest diameter and density of the tumor	CR, PR, SD, PD	Comprehensively consider the size and density of the tumor	It cannot be directly obtained by film reading; it has a high degree of subjectivity and is mainly used for gastric stromal tumors

## 2 Pathological tumor evaluation methods

### 2.1 Huvos grading system

The Huvos grading system is used to evaluate the pathological response of malignant tumors after treatment. It was proposed by Huvos et al. in 1977. Primarily, it is used to determine the degree of tumor necrosis in malignant tumors following treatments such as chemotherapy, thereby providing crucial evidence for assessing treatment efficacy, predicting prognosis, and guiding subsequent therapies (33). The Huvos grading system determines the tumor grade based on the area ratio of the necrotic area and the viable tumor cell area in the surgically resected tumor specimen. It can be classified into four grades. Grade I indicates that almost no tumor necrosis caused by chemotherapy is observed. Grade II implies that chemotherapy is mildly effective, with a tumor tissue necrosis rate of >50%, and viable tumor tissue is still present. Grade III indicates that chemotherapy is partially effective, with a tumor tissue necrosis rate of >90%, and residual viable tumor tissue can be seen in some tissue sections. Grade IV indicates that no viable tumor tissue is observed in any of the tissue sections.

The Huvos grading system is an assessment method based on the pathological examination of tumor tissue specimens obtained through surgical resection. In clinical practice, pathological examination is one of the routine diagnostic methods; therefore, it is relatively easy to perform without the need for additional complex equipment or techniques. The Huvos grading system can accurately reflect the actual effect of neoadjuvant therapy on tumors and provide a strong basis for adjusting subsequent treatment plans. The higher the score, the higher the degree of tumor necrosis and the better the treatment effect. For patients with a high score, the intensity of subsequent treatment can be reduced or more conservative treatment methods can be selected, whereas for patients with a low score, strengthening treatment or changing the treatment plan needs to be considered. The Huvos grading system can also reflect the survival rate of patients. Patients with a high score usually have a better prognosis, lower recurrence risk, and longer survival periods. This enables us to make a preliminary judgment on the prognosis of patients in the early stage of treatment.

However, the Huvos grading system has some limitations. The Huvos grade is mainly evaluated based on the pathological results after surgery, and the time point of evaluation is fixed, which cannot reflect the dynamic change process of the tumor during treatment. Moreover, it cannot be evaluated in patients who are ineligible for surgery. Although the Huvos grading system has relative objectivity, there is still subjectivity in the acquisition and preparation of pathological specimens as well as in score determination among different pathologists. In addition, incomplete or poor-quality pathological specimens obtained after surgery can affect the final results.

### 2.2 Miller–payne grading system

The Miller–Payne grading system, which was proposed by Miller and Payne et al. in 1989, evaluates the pathological response of breast cancer after neoadjuvant therapy. Unlike the Huvos grading system, the Miller–Payne grading system is based on the changes in the number and density of tumor cells before and after neoadjuvant therapy (34). The Miller–Payne criteria can be divided into five grades: G1: compared with before treatment, the number and distribution of tumor cells remain largely unchanged after treatment; G2: a mild reduction in tumor cell density and a decrease in the number of tumor cells occur, but the reduction does not exceed 30%; G3: a reduction in tumor cell density between 30% and 90% occurs; G4: reduction in tumor cell density exceeds 90%, and most tumor cells are killed, with only small clusters or single tumor cells remaining; G5: the tumor completely disappears with no residual invasive carcinoma; however, ductal carcinoma *in situ* may be present.

According to the Miller–Payne criteria, different grades clearly reflect the effects of neoadjuvant therapy. For patients with a higher grade, a reduced resection range can be considered during surgery. Conversely, for patients with a lower grade, the surgical approach may need to be re-evaluated, and a wider resection range should be considered to ensure the maximum removal of tumor tissue and reduce the risk of local recurrence.

The drawbacks of this grading system are as follows. First, it only evaluates changes in the primary tumor and does not consider lymph node metastasis. Second, there may be deviations in cases where the tumor density is uneven. Finally, most pathological specimens before neoadjuvant chemotherapy are obtained through needle biopsy, and the local pathology obtained through needle biopsy may not be able to represent the overall pathology of the tumor.

### 2.3 Residual cancer burden assessment criteria

The RCB assessment criteria are also a pathological evaluation method for the efficacy of neoadjuvant therapy in breast cancer; these criteria were proposed by the MD Anderson Cancer Center in the United States in 2007. They comprehensively consider multiple factors such as the extent, density, and proportion of the residual tumor in the primary breast cancer focus; number of positive lymph nodes; and maximum diameter of metastatic carcinoma in lymph nodes to quantify the status of the residual tumor. These criteria consider more comprehensive factors than the Miller–Payne criteria; therefore, they are currently more clinically recommended for evaluating the efficacy of neoadjuvant therapy in breast cancer. The RCB assessment criteria input data, such as the extent of the residual tumor in the primary breast focus, cell density of the residual tumor, proportion of *in situ* carcinoma, number of positive lymph nodes, and maximum diameter of residual metastatic carcinoma in lymph nodes, into an online calculator (www.mdanderson.org/breastcancer_RCB) to calculate the corresponding score. The RCB criteria classify cancers into four grades. RCB-0 indicates that the breast cancer tissue has completely disappeared or that there is only minimal residue. RCB-I indicates that breast cancer tissue has been decreased, but there is still a large amount of residue. RCB-II indicates that the tumor has a PR to treatment, and there is still a moderate amount of residue. RCB-III indicates that the tumor is resistant to chemotherapy, with extensive residue, and the breast cancer cells have hardly changed or have become more severe. The lower the grade, the better the treatment effect.

The RCB assessment criteria, as a continuous numerical value, can more precisely evaluate the amount of tumor residue in patients with breast cancer after receiving neoadjuvant therapy. For patients with breast cancer who have not achieved pathological CR, the RCB assessment criteria can be further refined to distinguish different degrees of tumor residue, thereby providing a more accurate basis for subsequent treatment decisions (35). Analytical studies have confirmed that the RCB assessment criteria can accurately predict the prognosis of patients (36); moreover, these criteria have a prognostic value and reflect treatment efficacy for different breast cancer subtypes (37). The RCB assessment criteria are closely related to the survival rate of patients with breast cancer. Researchers from the MD Anderson Cancer Center at the University of Texas conducted a pooled analysis of >5100 patients with breast cancer and found that the RCB assessment score had an independent and strong prognostic effect on all breast cancer phenotypes. For every one-unit increase in the RCB score, the risk of recurrence, metastasis, or death in patients with breast cancer of all subtypes increased by 82%. The greater the residual tumor, the higher the RCB assessment score and the higher the risk of distant recurrence. For different types of tumors, the survival rate after CR also varies. The 5-year survival rate of patients with breast cancer after CR can reach over 90%.

However, the RCB assessment criteria have some limitations. They require multiple parameters, and calculating the score is time-consuming. The evaluation process is subjective as different physicians may interpret the parameters differently. The RCB assessment criteria mainly focus on the evaluation of primary lesions and lymph nodes and fail to correctly evaluate the efficacy when the tumor invades certain specific locations (e.g., skin). Above mentioned pathological tumor evaluation methods could been seen in **Table 2**.

**Table 2:** Basic information, advantages, and disadvantages of pathological evaluation methods.

Evaluation criteria	Proposed time	Measurement method	Grading criteria	Advantages	Disadvantages
Huvos Grading System	1977	Proportion of the area of necrotic regions to that of viable tumor regions in tumor tissue	Grade I indicates almost no observable tumor necrosis; Grade II means the tumor necrosis rate is >50%; Grade III implies the tumor necrosis rate is >90%; and Grade IV indicates no viable tumor tissue is observed	It is easy to obtain and can accurately reflect the effect of neoadjuvant therapy	It cannot reflect the dynamic changes of the tumor and is subject to subjectivity
Miller–Payne Grading System	1989	Percentage of tumor cells	G1 indicates that there is no change in tumor cells; G2 indicates that the reduction in tumor cells does not exceed 30%; G3 indicates that the reduction in tumor cells is between 30% and 90%; G4 indicates that the reduction in tumor cells exceeds 90%; and G5 indicates that the tumor has completely disappeared	It can be used to determine the intensity of postoperative chemotherapy and radiotherapy	Lymph node metastasis situation is not taken into account
RCB Assessment Criteria	2007	Range and density of residual tumor in the primary focus and positive lymph nodes	Grade 0 indicates that the breast cancer tissue has completely disappeared. Grade I indicates that a reduction in the breast cancer tissue is observable. Grade II indicates that the tumor shows a PR to treatment, with moderate residue remaining. Grade III indicates that the tumor is resistant to chemotherapy, with extensive residue present	Precisely evaluate the amount of tumor residue after neoadjuvant therapy; it has prognostic value for all breast cancer subtypes	There is an excessive number of parameters, and it is highly subjective

## 3 Summary

Neoadjuvant therapy for malignant tumors has become a routine tumor treatment method. Currently, the commonly used evaluation methods for neoadjuvant therapy include the WHO criteria, RECIST criteria, and Huvos grading system. However, for tumors with nonuniform regression or reduced local activity, the current evaluation methods are limited. Therefore, a large number of clinical trials is needed to further verify and support the use of efficient and accurate emerging technologies to evaluate the efficacy of neoadjuvant therapy. Artificial intelligence (AI) is a field of technology and science that enables computer systems to simulate human intelligent behavior (38, 39). Its core objective is to enable machines to perform tasks that typically require human intelligence. In recent years, it has been widely applied across multiple disciplines (40–44).

Future research on evaluation methods for neoadjuvant tumor therapy should focus on integrating multimodal data fusion with AI technology. Deep learning-based radiomics can automatically analyze imaging features from CT, MRI, and PET-CT (45–48); extract quantitative data on tumor morphology, texture, and metabolism; and integrate clinical data to construct predictive models for more accurate assessment of tumor regression and treatment response (49). In addition, the application of AI in the field of pathology, such as whole slide image analysis, can assist the Huvos score or RCB criteria. By identifying tumor necrosis areas and quantifying changes in cell density, AI can minimize human errors and enhance evaluation efficiency (50–52). AI can also integrate multisource data, including electronic medical records and genomics, using natural language processing technology. This integration holds promise for individualized efficacy prediction and dynamic monitoring. AI technology holds great potential. However, challenges, such as data standardization, algorithm interpretability, and ethical problems, must be addressed. Through interdisciplinary collaboration and prospective research, AI-driven intelligent evaluation systems could become next-generation core tools for assessing neoadjuvant tumor therapy, thereby advancing the development of precision medicine.
